# Assessing Cognitive Workload in Motor Decision-Making through Functional Connectivity Analysis: Towards Early Detection and Monitoring of Neurodegenerative Diseases

**DOI:** 10.3390/s24041089

**Published:** 2024-02-07

**Authors:** Leonardo Ariel Cano, Ana Lía Albarracín, Alvaro Gabriel Pizá, Cecilia Elisabet García-Cena, Eduardo Fernández-Jover, Fernando Daniel Farfán

**Affiliations:** 1 Neuroscience and Applied Technologies Laboratory (LINTEC), Bioengineering Department, Faculty of Exact Sciences and Technology (FACET), National University of Tucuman, Superior Institute of Biological Research (INSIBIO), National Scientific and Technical Research Council (CONICET), Av. Independencia 1800, San Miguel de Tucuman 4000, Argentina; 2 ETSIDI-Center for Automation and Robotics, Universidad Politécnica de Madrid, Ronda de Valencia 3, 28012 Madrid, Spain; cecilia.garcia@upm.es; 3 Institute of Bioengineering, Universidad Miguel Hernández of Elche, 03202 Elche, Spain; 4 Research Networking Center in Bioengineering, Biomaterials and Nanomedicine (CIBER-BBN), 28029 Madrid, Spain

**Keywords:** neurodegenerative diseases, cognitive workload, statistical modeling, motor planning, decision-making, functional connectivity

## Abstract

Neurodegenerative diseases (NDs), such as Alzheimer’s, Parkinson’s, amyotrophic lateral sclerosis, and frontotemporal dementia, among others, are increasingly prevalent in the global population. The clinical diagnosis of these NDs is based on the detection and characterization of motor and non-motor symptoms. However, when these diagnoses are made, the subjects are often in advanced stages where neuromuscular alterations are frequently irreversible. In this context, we propose a methodology to evaluate the cognitive workload (CWL) of motor tasks involving decision-making processes. CWL is a concept widely used to address the balance between task demand and the subject’s available resources to complete that task. In this study, multiple models for motor planning during a motor decision-making task were developed by recording EEG and EMG signals in n=17 healthy volunteers (9 males, 8 females, age 28.66±8.8 years). In the proposed test, volunteers have to make decisions about which hand should be moved based on the onset of a visual stimulus. We computed functional connectivity between the cortex and muscles, as well as among muscles using both corticomuscular and intermuscular coherence. Despite three models being generated, just one of them had strong performance. The results showed two types of motor decision-making processes depending on the hand to move. Moreover, the central processing of decision-making for the left hand movement can be accurately estimated using behavioral measures such as planning time combined with peripheral recordings like EMG signals. The models provided in this study could be considered as a methodological foundation to detect neuromuscular alterations in asymptomatic patients, as well as to monitor the process of a degenerative disease.

## 1. Introduction

Currently, there is a growing acceptance of the notion that neurodegenerative diseases (NDs) can be better characterized in terms of the functional and anatomical alterations of neuromuscular connectivity [1,2]. Numerous studies have conclusively demonstrated the presence of widespread changes in cerebral connectivity throughout the neurodegenerative processes of diseases such as Alzheimer’s [3], Parkinson’s [4], amyotrophic lateral sclerosis [5], and frontotemporal dementia [6]. These alterations often give rise to motor symptoms, such as tremors, muscular rigidity, bradykinesia, as well as non-motor symptoms, including cognitive impairment, emotional changes, and sleep disorders, among others [7,8,9]. Taking into consideration the neuroanatomical structures that these diseases can alter, it becomes evident that the execution of motor tasks can also be affected.

In general, the execution of complex motor actions involving processes such as planning, decision-making, and execution, entails a certain cognitive workload (CWL), engaging the involvement of multiple brain areas and processes related to the coordinated activation of muscles. Consequently, alterations in any part of the neuromuscular network that governs such motor programs can lead to inefficiencies in their execution [10]. In this context, signals such as the electroencephalogram (EEG) and the electromyogram (EMG) have been demonstrated to be promising tools for determining and quantifying cortical and motor functional states associated with motor program execution. Furthermore, they could potentially serve as tools for the early detection of neurodegenerative diseases [11].

CWL represents the balance between task demand and the subject’s available resources to complete it [12,13]. Individuals with different experiences and abilities may complete the same task in diverse manners. Previous research has shown that EEG measurements can be used to classify the levels of CWL. Recent studies using EEG have introduced quantitative models for estimating CWL using spectral features [14,15,16,17] and brain networks [18,19,20]. Chikhi et al. [21] have suggested that it is crucial to explore the relation between CWL and other factors that may potentially influence EEG spectral power and to combine this measure with other processing methods for the central and peripheral nervous system. This multidimensional approach will provide a more-comprehensive understanding of the complex dynamics underlying CWL.

In the field of motor control, there is a growing interest in evaluating performance during the motor decision-making (MDM) process [22,23]. In the literature, reaction time (RT) is a widely used measurement for assessing performance [24,25,26,27]. In a previous work [28], we have shown that MDM during reaction tasks requires a remarkably short time, less than 500 ms. In this context, electrophysiological signal recordings (i.e., EEG, EMG) provide high temporal resolution, enabling the neural activity to be processed in short time intervals. Signal coupling is a valid method to assess functional connectivity between the brain and muscles or between muscles. Measurements such as corticomuscular coherence (CMC) and intermuscular coherence (IMC) are employed [29]. Recently, coherence analysis using a new pre-processing computational technique [30] combining time–frequency analysis and the continuous wavelet transform has been introduced. This method enables processing data recorded during large joint or multi-joint movements [31,32,33,34,35,36,37]. There is still divergence in the results reported and the consensus regarding the specific frequency bands associated with motor control [38]. Our study builds upon emerging evidence [31,32,33,34,35,36,39] suggesting a relationship between beta-band CMC and the motor command direction from the cortex to the muscle. It is well known that motor production is a complex process involving the structural and functional interconnections between several brain regions and the peripheral system. Previous studies [23,40,41,42,43] have suggested that certain aspects of motor planning take place in the premotor area (PMA) and supplementary motor area (SMA). These regions are believed to play crucial roles in coordinating and orchestrating motor programs and actions. Evidence suggests that MDM can be assessed by monitoring the frontal brain cortex. However, the implicated networks remain unclear [44]. Despite other studies [34,37,45,46,47] having previously employed functional connectivity states (i.e., CMC) to identify differences in motor control between healthy subjects and patients with motor disorders, this study introduces an approach for monitoring functional connectivity changes in the beta band to analyze the relationship between MDM and CWL. In order to explore this relationship, we designed an experimental protocol based on a go/no-go task.

In this study, we assessed MDM performance and functional connectivity to develop models using multiple linear regression. Likewise, to address CWL in central processing, the performance of the models was compared during a motor reaction task. Although this study was applied to healthy subjects, it is intended that this modeling methodology be applied to subjects with ND in order to characterize, monitor, and predict their neurodegenerative processes.

## 2. Materials and Methods

### 2.1. Participants

Seventeen healthy volunteers (nine males, eight females, age 28.66 ± 8.8 years) participated in a motor reaction study using visual stimulus. The study was conducted by following the ethical guidelines established in the Declaration of Helsinki and approved by the ethics committee of the Miguel Hernandez University from Elche, Spain (Reference: IB.EFJ.04.21). Subjects were previously instructed about the tasks and gave written informed consent. All participants self-reported as right-handed, with normal or corrected-to-normal vision, and declared no history of neurological or locomotor disorders.

### 2.2. Experimental Design

**Figure 1:**
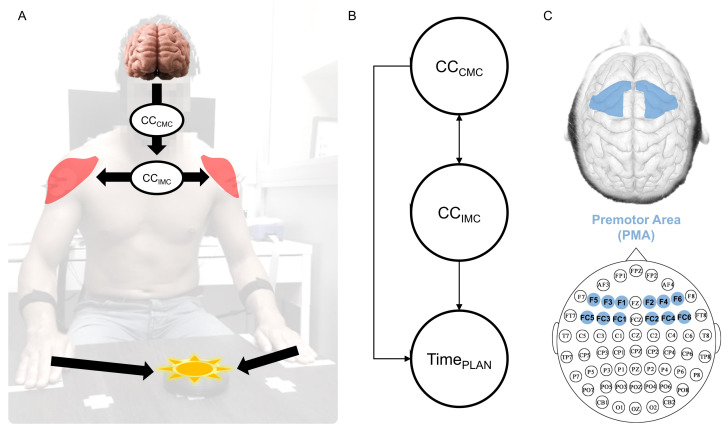
(**A**) Experimental setup. (**B**) Variables’ interaction for modeling. (**C**) Sixty-four-channel EEG configuration system; electrodes in blue represent the premotor area.

The experiment consisted of sitting the subjects in front of a table with their hands located in a predefined position (Figure 1A). On the table and in front of the subject, a device was placed (reactimeter). The reactimeter emits programmed lights following a predefined protocol. The device emits one-color light (red or green) and includes a motion sensor. When the subject places the hand on the reactimeter, the light turns off; then, it is possible to measure the time lapse between the light being turned on and turned off. The subject was instructed to associate the green color stimulus with the right hand movement and the red color with the left hand movement. The task consisted of randomly emitting green or red light, so the subject had to make a decision before moving any hand. Twenty repetitions were performed in this condition. The minimum time between repetitions had to be 6 s; after that, the stimulus was spontaneously delivered. Participants were unaware of this information and were warned to remain attentive. In order to avoid artifacts in EEG recordings due to hand movements, the device was located 30 cm from the subject’s hands. During the test, the participant should keep the gaze fixed on the device.

### 2.3. Instrumentation

A linear position transducer with a 1 kHz sampling rate (WinLaborat, Buenos Aires, Argentina), was attached to both forearms using adjustable tapes in order to detect the hands’ movement. EMG surface recordings were acquired by an RHA2000-series (Intan, Los Angeles, CA, USA) acquisition system (16-channel amplifier and 25 kHz sampling rate). Muscle activity data were collected from both anterior deltoid muscles using Ag/AgCl button electrodes (Dormo, Barcelona, Spain); skin surface preparation and electrodes’ placement were performed following the SENIAM guidelines. To measure the RT, we used a system designed and manufactured in our laboratory (LINTEC, San Miguel de Tucuman, Argentina). It consists of a control center programmed for eliciting the signal via WiFi to the device to turn on/off the light. We used the SynAmps RT system, a 1 kHz sampling rate, and a 64-channel Quik-Cap helmet (Compumedics Neuroscan, Char lotte, NC, USA) for cortical activity recordings. EEG data were acquired with software Curry v7. Conductive gel was applied to the helmet electrodes, ensuring an impedance <25 kΩ. An external synchronization system (trigger) was activated by the operator before the test began. The trigger was configured to simultaneously send a signal to both the Intan and SynAmps systems. Both recordings were acquired separately, but synchronized later by signal pre-processing. The position transducer and the reactimeter data were acquired by using Intan board’s auxiliary channels.

### 2.4. Signal Pre-Processing

The analysis was performed offline with the Matlab 2020b software. The motion capture signal was analyzed using classical kinematics formulations for obtaining displacement data. The reactimeter data were kept as binary on–off data. Electromyography recordings were resampled at 1 kHz. We applied a 5th-order Butterworth filter (band-pass 13–100 Hz, band-stop 49–51 Hz). Electroencephalography signals were pre-processed in two stages. In the first stage, the EEGLAB toolbox [48] was used. The channels were re-referenced to the average, and an FIR high-pass 4 Hz filter was applied. Next, a visual inspection using the ‘scroll data’ and ‘channel properties’ functions corroborated no big artifacts coming from non-typical biological sources. However, as Klug and Gramann [49] recommended, we avoided eliminating channels or segments of the recording that contain artifacts of apparent biological origin (muscle contractions, eye-blinking, eye movement) before Independent Component Analysis (ICA). The next step was to perform ICA to identify signal components that could be covering the cortical signal. The RunICA algorithm was applied on 64 channels. Next, the ICLabel algorithm was applied to classify Independent Components (ICs). ICs identified as ‘muscle’, ‘eye’, ‘bad channel’, and ‘heart’ with a probability higher than 0.6 were labeled. After a visual inspection of each IC, some of them were discarded. We discarded 19.97 ± 5.72 from 64 ICs per series. Finally, the signal was reconstructed without the discarded components. The second stage was performed in Matlab, and a fifth-order Butterworth filter (band-pass 13–100 Hz) was applied.

### 2.5. Motor Planning Phase Identification

Specific events during the task (i.e., light turn-on, muscle contraction onset) were used to determine the onset and finalization of the motor decision-making phase. The time interval between the light turning on and the muscle contraction onset was named the ‘motor planning phase’ (PLAN). PLAN represents the time interval when the subject makes a decision about which hand should move and planning its movement. Visual inspection was conducted to discard execution mistakes, and 3% of the repetitions of the right hand and 3.1% of the left hand were discarded. Motor planning phase durations $\left(Time_{PLAN}\right)$ were calculated for the remaining repetitions. A more-detailed explanation of event detection can be seen in the Supplementary Material.

### 2.6. Functional Connectivity Computation

The procedure to compute CMC and IMC is based on the method of Bigot et al. [30]. We made minor variations in order to analyze events in a fixed time window. This procedure can be summarized in the next steps: (1) select two time series (i.e., EEG-EMG or EMG-EMG); (2) select the segment to analyze (we used a 1 s window prior to muscle contraction onset); (3) apply the continuous wavelet transform of both segmented signals; (4) compute the ‘Mean Cross-Spectrum’ between each signal pair, and compute the ‘Mean Auto-Spectrum’ for both signals separately; (5) compute the ‘Magnitude-Squared Coherence’ only where the ‘Mean Cross-Spectrum’ was statistically significant; (6) compute the CMC as the single value (mean) within each window of interest. The CMC was computed between an EMG signal from the anterior deltoid muscle of the moved limb and 64 EEG channels (Figure 1C). The IMC was computed between EMG signals from both anterior deltoid muscles. For computing the CMC and IMC in the PLAN phase, we used a 1 s window prior to muscle contraction onset. The limits in the frequency for the window were established between 15 and 30 Hz, corresponding to the beta-band. For calculating the connectivity change (CC) within the same fixed time window, we divided this 1 s window into two 500 ms windows. Detailed explanations with a step-by-step graphical example can be seen in the Supplementary Material.

We calculated the CC for the CMC as indicated by Equation (1); it was also applied to the CC for the IMC (Equation (2)). According to Equations (1) and (2), if the change is positive, it means ‘synchronization’, and if the change is negative, ‘desynchronization’. Six EEG channels were selected to represent the PMA: F1, F3, F5, FC1, FC3, and FC5 (left hemisphere) and F2, F4, F6, FC2, FC4, and FC6 (right hemisphere). The $CC_{CMC}$ data extracted from these six channels (highlighted in blue in Figure 1C) for each brain hemisphere were pooled to obtain a single value as the mean.

(1) $$CC_{CMC}=\frac{CMC_{\mathrm{final}}-CMC_{\mathrm{initial}}}{CMC_{\mathrm{initial}}}\times 100$$

(2) $$CC_{IMC}=\frac{IMC_{\mathrm{final}}-IMC_{\mathrm{initial}}}{IMC_{\mathrm{initial}}}\times 100$$

### 2.7. Statistical Procedure for Modeling and Testing

**Figure 2:**
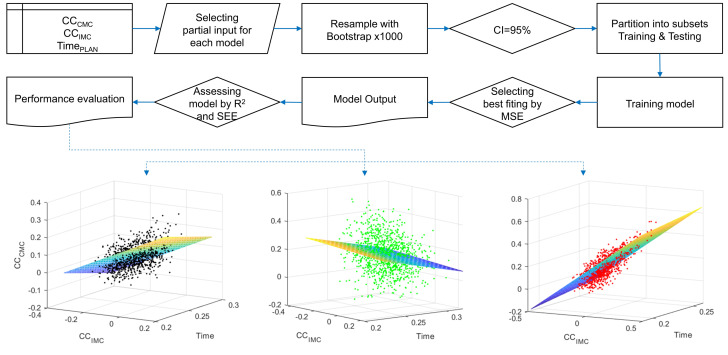
Modeling procedure flowchart. In the upper part, each step of the modeling procedure. In the lower part, three-dimensional representations of the resampled data: for the bilateral model (black dots), for the right model (green dots), for the left model (red dots). The colored planes represent the developed models.

The normal distribution of the data was verified through the Shapiro–Wilk test only for the variable $Time_{PLAN}$. The results are presented as the means and standard deviations. To test the hypothesis, we performed a *t*-test for paired samples. For the variables $CC_{CMC}$ and $CC_{IMC}$, a normal distribution was not verified. The results are presented as the medians. We used the non-parametric Wilcoxon one-tailed Signed-Rank test for paired comparisons. For the models’ formulation, we assigned $CC_{CMC}$ as the dependent variable (*y*) and $Time_{PLAN}$ ($x_{1}$) and $CC_{IMC}$ ($x_{2}$) as the independent variables, as indicated by Equation (3).

(3) $$y=a_{1}x_{1}+a_{2}x_{2}+c$$

where $a_{1}$ and $a_{2}$ are the coefficients for independent variables $x_{1}$, $x_{2}$; *c* is the intercept. Three models were calculated as follows: $M_{RIGHT}$ includes the data associated with the right hand election. $M_{LEFT}$ includes the data associated with the left hand election. $M_{BILATERAL}$ includes all the data. For each model, the same procedure was applied using Matlab 2020b, depicted in Figure 2.

The variables of interest were randomly resampled using the bootstrap function within the 95% confidence interval. Resampling was performed, obtaining 1000 samples excluding extreme outlier values. Subsequently, a randomized partition algorithm was applied to create subgroups using the cvpartition function with the following parameters: nObservations = 1000, ‘kFold’, nFolds = 100. cvpartition is a Matlab built-in function to define training and test sets for validating a statistical model using cross-validation. One hundred partitions were obtained for each model. The models were trained using cross-validation (crossval function) with the corresponding k-folds. The model with the lowest mean-squared error (MSE) was selected as the most-appropriate in each case. Finally, to evaluate and compare the performance of the selected models, fitting descriptive measures were used: the determination coefficient ($R^{2}$) and the standard error of estimation (SEE). $R^{2}$ represents the proportion of the dependent variables’ variability explained by the model, and SEE represents the variability of the model’s prediction errors. Therefore, $R^{2}$ with a high value and SEE with a low value indicate that the model is able to make accurate predictions.

## 3. Results

The results for connectivity changes ($CC_{CMC}$, $CC_{IMC}$) and planning time ($Time_{PLAN}$) are presented in Table 1. $CC_{CMC}$ represents functional connectivity changes in central nervous processing. $CC_{IMC}$ represents functional connectivity changes in the peripheral system. $Time_{PLAN}$ represents performance in motor decision-making.

**Figure 3:**
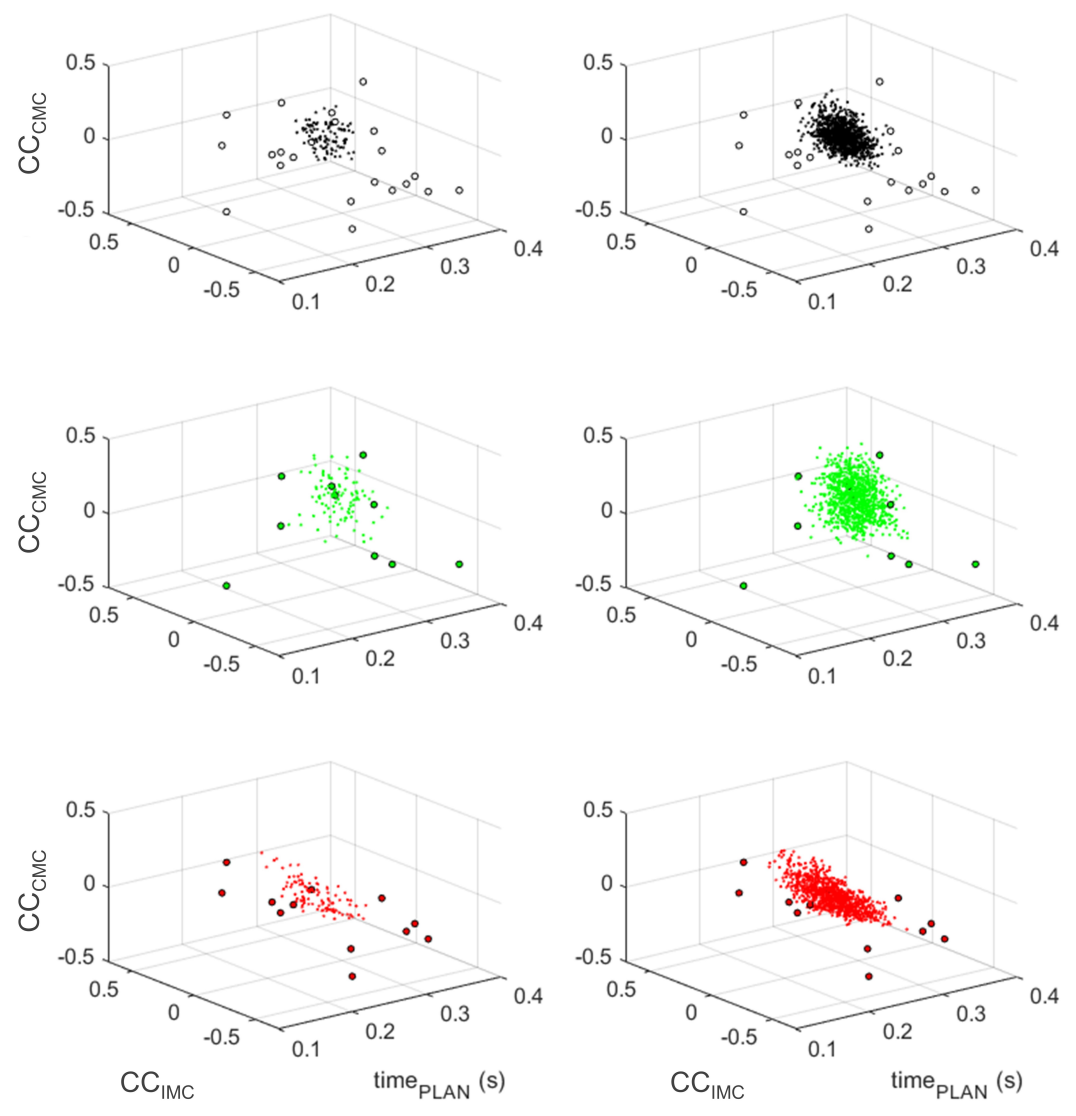
Distribution of resampled data using the bootstrap procedure. The original data are represented by circles (o), while the resampled data are represented by dots (·). In the left column, ×100 resampling; in the right column, ×1000 resampling. In the top row, data selection for the bilateral model (in black); in the middle row, data for the right hand model (in green); in the bottom row, data for the left hand model (in red).

Firstly, we conducted a temporal analysis. $Time_{PLAN}$ corresponding to the right hand movement was significantly higher than the left hand (*p* < 0.05). Secondly, comparisons for connectivity measures between hands were conducted. $CC_{CMC}$ and $CC_{IMC}$ did not show significant differences. The distribution of the original and resampled data using the bootstrap procedure in the three dimensions of the models is presented in Figure 3. To better visualize the trends in the data distribution, two resamplings (×100 and ×1000) are depicted.

**Figure 4:**
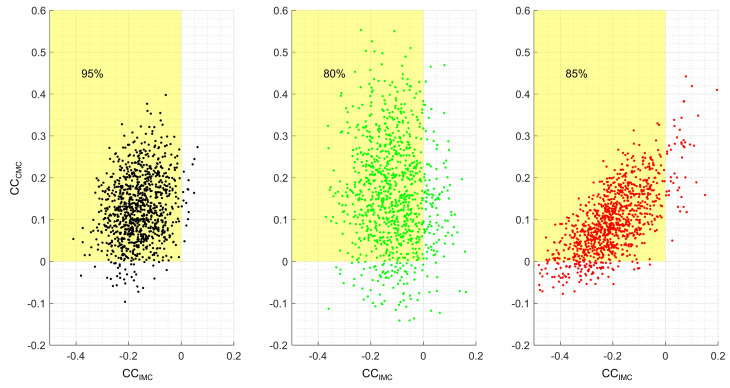
Distribution of resampled data according to the connectivity variables. The shaded rectangle (in yellow) represents the quadrant formed by positive values for the corticomuscular connectivity variable ($CC_{CMC}$) and negative values for the intermuscular connectivity variable ($CC_{IMC}$). In the left figure, resampled data for the bilateral model are presented (in black), with 95% of the data in the expected quadrant. In the middle figure, resampled data for the right hand model are shown (in green), with 80% of the data in the expected quadrant. In the right figure, resampled data for the left hand model are displayed (in red), with 85% of the data in the expected quadrant.

Figure 4 depicts the trend of the resampled data distribution in the connectivity variables. $CC_{CMC}$ data predominantly assumed positive values (synchronization), in line with our previous expectations. Additionally, it was observed that the $CC_{IMC}$ data predominantly assumed negative values (desynchronization), since a differentiation between muscles was expected based on the exclusive selection of one muscle over the other during decision-making. The data did not follow these patterns, which was attributed to the heterogeneity of the original samples. Overall, for $M_{BILATERAL}$, 95% of the data remained within the expected quadrant, while for $M_{RIGHT}$, 80%, and for $M_{LEFT}$, 85%.

**Figure 5:**
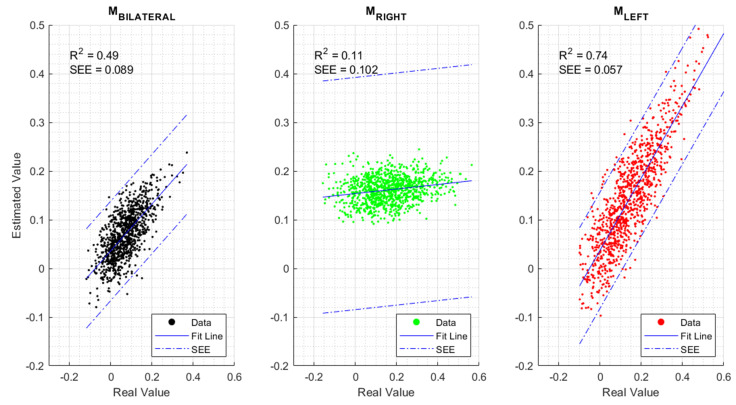
Scatter plot of model fit. The X-axis represents the values of the original data for $CC_{CMC}$; the Y-axis represents the model outputs. In the left figure, the fit distribution for $M_{BILATERAL}$ is depicted in black; in the middle figure, $M_{RIGHT}$ is represented in green; in the right figure, $M_{LEFT}$ is shown in red. The solid lines (-) represent the fitted line; dashed and dotted lines (-·-) represent the limits of the standard error of estimation (SEE).

The results of the three models are presented by Equations (4)–(6), while Figure 5 visually illustrates the performance of each model.

(4) $$M_{BILATERAL}:y=-3.2105x_{1}+0.075775x_{2}+0.86281,$$

(5) $$M_{RIGHT}:y=-2.657x_{1}-0.012588x_{2}+0.91984,$$

(6) $$M_{LEFT}:y=0.9242x_{1}+0.73318x_{2}+0.02245,$$

where *y* is the dependent variable $CC_{CMC}$; $x_{1}$ is the independent variable $Time_{PLAN}$; $x_{2}$ is the independent variable $CC_{IMC}$.

**Figure 6:**
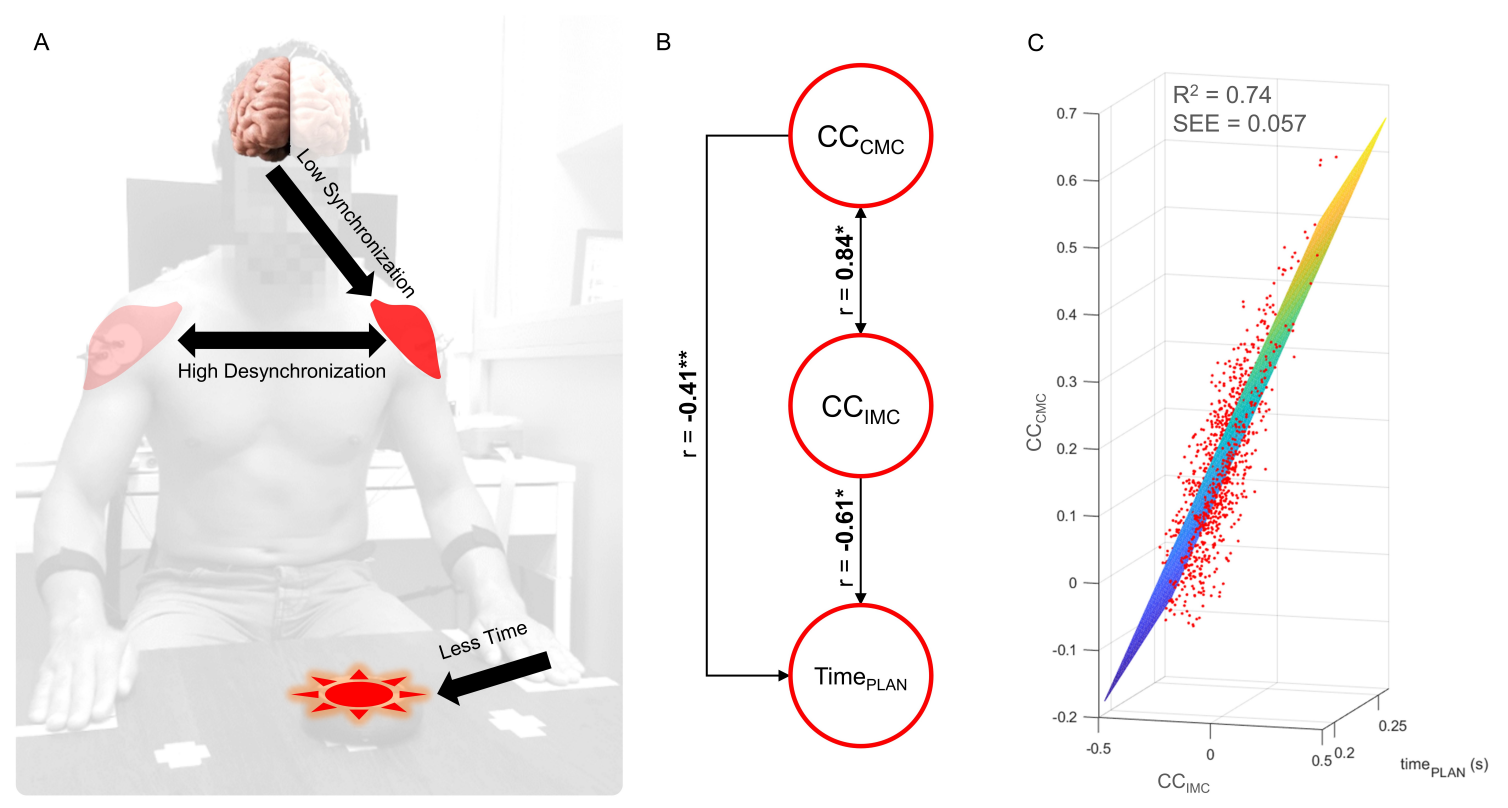
Graphical representation of the left hand motor planning model. (**A**) The right (contralateral) premotor area synchronizes with the left anterior deltoid muscle (agonist muscle); both anterior deltoid muscles desynchronize between them; the motor planning time is shorter for the left hand compared to the right hand. (**B**) Strong correlation (0.84) between connectivity changes. Moderate and inverse correlation between corticomuscular synchronization (−0.41) and planning time, as well as between intermuscular desynchronization (−0.61) and planning time. (**C**) Three-dimensional representation of the fitting plane of the left hand model ($M_{LEFT}$), showing the distribution trend and high predictive capacity. * *p* < 0.01 ** *p* < 0.05.

The descriptive measures shown in Figure 5 revealed that $M_{BILATERAL}$ showed $R^{2}$ = 0.49, indicating that the model was able to explain 49% of the observed variability in the dependent variable, leaving 51% of unexplained variability, which may be attributed to other variables not included in the analysis or random factors. $M_{LEFT}$, with $R^{2}$ = 0.74, demonstrated a high percentage of explained variability and the lowest SEE, while $M_{RIGHT}$, with $R^{2}$ = 0.11, indicated that the model poorly explained the variability based on the selected independent variables, consequently showing the highest SEE. Lastly, based on the high accuracy of $M_{LEFT}$, Pearson correlation coefficients were computed between $CC_{CMC}$ and $CC_{IMC}$ (r = 0.84, *p* < 0.01), between $CC_{CMC}$ and $Time_{PLAN}$ (r = −0.41, *p* < 0.05), and between $CC_{IMC}$ and $Time_{PLAN}$ (r = −0.61, *p* < 0.01). Finally, Figure 6 summarizes the performance of $M_{LEFT}$.

## 4. Discussion

In this study, three models were developed to estimate the corticomuscular connectivity change in the contralateral premotor area ($CC_{CMC}$) using two independent variables: planning time ($Time_{PLAN}$) and intermuscular connectivity change ($CC_{IMC}$). Dissimilar results obtained from the models suggest that the central processing for both hands involves different resources. Consequently, the cognitive workload (CWL) for decision-making between two hands has shown disparities.

According to Cohen’s classification [50] in relation to the determination coefficient, the results showed that the $M_{BILATERAL}$ model had a moderate predictive capacity ($R^{2}$ = 0.49, SEE = 0.089). This indicates that $M_{BILATERAL}$ is non-specific, as nearly half of the variability in the model output remained unexplained. Currently, some postulates [51,52,53] about hemisphere specializations are discussed in the literature. A recent study [52] has shown activation on the left hemisphere (parietal and frontal regions) during movements of either the left or the right hand. In contrast, the activation of the same regions in the right hemisphere was limited to movements with the left hand (contralateral). This suggests that the left hemisphere performs some extra work. In the case that the left hand movement has to be suppressed, the activity of both hemispheres will be disrupted. In addition, the processing time required for both hemispheres’ activation increases. This is not the case for right hand movement, in which only one hemisphere is activated [52]. These previous studies based their conclusions about hand performance on RT measures. In our previous work [28], we used the planning time and showed the opposite result. The planning process for the left hand movement required significantly less time than the right hand in right-handed subjects, as we have shown. This increased planning time for right hand movement reveals that the reactive inhibition of the left hand (resulting in a right hand selection) requires a longer processing time compared to the opposite decision (left hand selection). The shorter processing time for the left hand could indicate a higher efficiency of the nervous system in motor planning, as reflected in the high precision of the $M_{LEFT}$ ($R^{2}$ = 0.74, SEE = 0.057) compared to the low predictive capacity of $M_{RIGHT}$ ($R^{2}$ = 0.11, SEE = 0.102). Supporting this idea, an inversely proportional relationship was obtained between $Time_{PLAN}$ and $CC_{CMC}$ (r = −0.41) and $CC_{IMC}$ (r = −0.61). This evidence suggests that the relationship between time and connectivity changes could be interpreted as an efficiency measure. This issue will be further analyzed in the following paragraphs.

According to $M_{LEFT}$, there was a high correlation (r = 0.84) between these connectivity variables ($CC_{CMC}$ and $CC_{IMC}$). To interpret this correlation, it is necessary to consider the data distribution presented in Figure 4 (right panel). When brain resources were less required ($CC_{CMC}$ tends to zero), there was greater intermuscular desynchronization (i.e., increased intermuscular differentiation). In other words, if cerebral processing is low, intermuscular differentiation is high. Conversely, when central processing is high, muscular desynchronization tends to zero. In summary, $M_{LEFT}$ showed that lower corticomuscular synchronization corresponds to higher intermuscular desynchronization, shorter planning time, and greater efficiency in the whole processing. In the case of the right hand, $M_{RIGHT}$ did not provide a conclusive explanation for the same relation between variables, although the same pattern was observed in the descriptive measures presented in Table 1. When higher brain resources were required (median synchronization of 14.5%), the intermuscular differentiation decreased (median desynchronization of 0.5%). In consequence, a longer $Time_{PLAN}$ for the right hand was obtained in comparison to the left hand (*p* < 0.05). This result suggests a lower efficiency for right hand processing. For these reasons, despite the motor task being similar, motor planning processing for both hands was different, and a higher CWL for the right hand was obtained. This difference in processing is a central issue when assessing motor-related actions in healthy subjects. However, these results should also be considered when designing unilateral (or bilateral) motor tasks for evaluating patients affected by neurodegenerative diseases with motor impairments.

The use of connectivity measures in assessing the efficiency of motor decision-making tasks represents a novel approach in the field. While previous studies have predominantly relied on measures such as reaction time (RT) and other behavioral indicators, using planning times could represent a significant breakthrough for addressing central processes. On the other hand, it has been suggested that corticomuscular connectivity states can reveal differences in motor control efficiency in individuals with neuromuscular impairments such as stroke [34,45,46] or spinal cord injuries [47]. In this sense, our exploration of connectivity changes provides valuable insights into the underlying neural processes during motor planning in healthy subjects. Finally, the proposed modeling methodology and the findings described in this study could be employed for both early detection and characterization of ND (i.e., observing differences compared to healthy subjects) and for monitoring and tracking their progression (i.e., analyzing the dynamics of the models).

## 5. Conclusions

In this study, we demonstrated that it is possible to model motor tasks involving decision-making processes through the direct relationship between neuromuscular connectivity changes. In healthy subjects, we have presented evidence suggesting that motor planning for both hands involves different central processing, resulting in distinct motor decision-making performances. Despite the task demand being equal for both hands due to identical motor tasks, the cognitive workload was higher for right hand movement, requiring different brain resources compared to the left hand. These results allowed us to hypothesize that modeling the corticomuscular dynamics involved in decision-making processes, which can be altered by neurodegenerative disorders in preclinical stages, may serve as the methodological foundation for diagnosing and monitoring such disorders.

## Figures and Tables

**Table 1:** Experimental group data. $Time_{PLAN}$ values are presented as the means. $CC_{IMC}$ and $CC_{CMC}$ values are presented as the medians.

	Right Hand			Left Hand		
**Subject**	$Time_{PLAN}$ **(s)**	$CC_{IMC}$ **(-)**	$CC_{CMC}$ **(-)**	$Time_{PLAN}$ **(s)**	$CC_{IMC}$ **(-)**	$CC_{CMC}$ **(-)**
P1	0.298	−0.186	0.451	0.244	−0.424	−0.402
P2	0.225	1.057	0.739	0.198	1.215	1.266
P3	0.271	−0.005	0.145	0.188	−0.132	0.023
P5	0.257	−0.317	0.969	0.154	0.057	0.315
P6	0.348	−0.668	−0.184	0.229	−0.508	−0.171
P7	0.148	0.019	−0.318	0.213	−0.132	0.022
P8	0.304	−0.387	−0.225	0.189	0.623	0.919
P9	0.245	0.167	−0.079	0.260	−0.570	0.155
P10	0.236	0.106	0.285	0.251	−0.052	0.054
P11	0.240	−0.628	0.330	0.165	−0.249	0.134
P13	0.319	0.168	0.040	0.302	−0.585	−0.061
P14	0.231	−0.070	0.213	0.347	0.653	0.957
P15	0.372	0.523	−0.078	0.278	−0.660	−0.061
P16	0.316	−0.174	−0.252	0.308	−0.662	−0.147
P17	0.224	−0.030	1.069	0.199	0.370	−0.047
P18	0.273	1.255	0.192	0.192	−0.154	−0.001
P19	0.266	1.919	−0.032	0.183	1.174	0.201
Mean	0.269	-	-	0.229	-	-
Median	-	−0.005	0.145	-	−0.132	0.023

## Data Availability

The data presented in this study are available upon request from the corresponding author. The data are not publicly available due to data protection policies practiced at our institution, which contain information that could compromise the privacy of research participants.

## Supplementary Materials

The following are available online at https://www.mdpi.com/article/10.3390/s24041089/s1. The supporting information includes three detailed explanations: Procedure for identifying motor planning phase; Statistical determination of planning times differences; and Procedure for computing signal coupling (Corticomuscular and Intermuscular Coherence). Additionally, Figure S1. Diagram showing all synchronized time-series; Figure S2. Statistical comparison of motor planning phase durations between hands; and Figure S3. Summary for CMC compute procedure.

## Abbreviations

The following abbreviations are used in this manuscript:

CC	connectivity change
CMC	corticomuscular coherence
CWL	cognitive workload
EEG	electroencephalography
EMG	electromyography
ICA	Independent Component Analysis
IMC	intermuscular coherence
MDM	motor decision-making
ND	neurodegenerative disease
PLAN	motor planning phase
PMA	premotor area
RT	reaction time
SMA	supplementary motor area
