# Supplementary material for: Assessing Cognitive Workload in Motor Decision-Making through Functional Connectivity Analysis: Towards Early Detection and Monitoring of Neurodegenerative Diseases
Source: Sensors (Basel). 2024 Feb 7;24(4):1089. doi: 10.3390/s24041089 (PMC10893317; doi:10.3390/s24041089)
Supplement: Supplementary file 1 [file sensors-24-01089-s001.zip › sensors-2549138-supplementary.pdf]

TITLE:

Assessing Cognitive Workload in Motor Decision-Making through Functional Connectivity Analysis: Towards Early Detection and Monitoring of Neurodegenerative Diseases

SUPPLEMENTARY MATERIAL:

Part 1. Procedure for identifying Motor Planning phase.

Specific events during the task (i.e. light turn-on, muscle contraction onset, hand movement onset) were used to separate different phases (Fig. S1). The time-interval between light turning-on and the muscle contraction onset was named 'Motor Planning phase' (PLAN). Then, we decided to use 1-second window before the muscle contraction onset for each repetition to compute Signal Coupling (i.e. CMC and IMC) as detailed below.

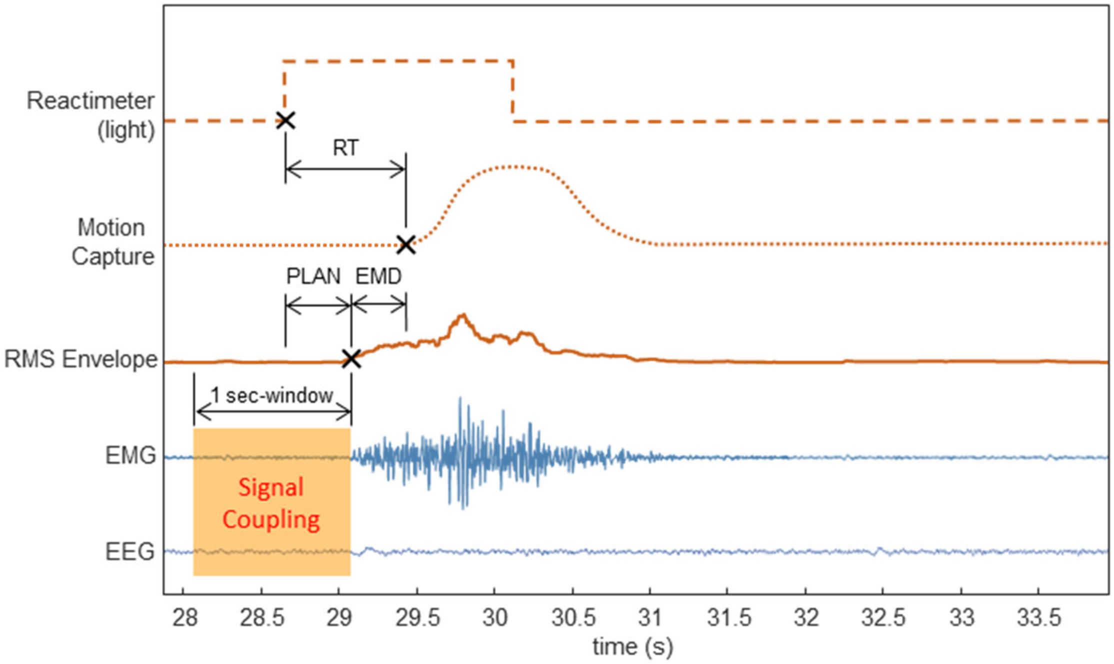

Figure S1. Diagram showing all synchronized time-series. Reaction Time (RT) is the sum of planning time (PLAN) and electromechanical delay (EMD). The crosses (x) indicate the onset of lights (dashed line), movement (pointed line), and muscle contraction (continuous line). The orange rectangle is a 1-second time-window prior to muscle contraction onset, starting before the light turn-on. PLAN phase is the interval delimited between light turn-on and effective muscle contraction detected. Signal coupling was computed between time-series (EMG, EEG) in this fixed-time window.

Determining the muscle contraction onset is crucial to separate planning phase (PLAN) phase from electromechanical delay (EMD). This determination was made in three steps. First, normalizing the

signal from the Reaction Time (RT) interval using the interval EMG max value, for each separated repetition. Second, detecting the envelopes by using the root mean square (RMS) of every repetition. Third, the onset of the effective contraction was determined as the envelope amplitude exceeded three standard deviations over the basal state, as we have previously described [2]. The muscle contraction onset is represented by a cross (X) in Figure S1. For the subsequent connectivity analyses, we used the unrectified and non-normalized EMG signal to meet both theoretical support and practical justification for coherence analysis [30].

Part 2. Statistical determination of $Time_{PLAN}$ differences

We conducted a temporal analysis comparing the hands performances. The normal distribution of the data was verified through the Shapiro-Wilk test only for the variable $Time_{PLAN}$. The results are presented in means and standard deviation. To test the hypothesis, we performed t-test for paired samples. The comparisons of planning phase duration revealed that $Time_{PLAN}$ corresponding to the right-hand movement was significantly higher than the left-hand ($p<0.05$). The figure S2 presents the normal distribution of time data and the significant differences.

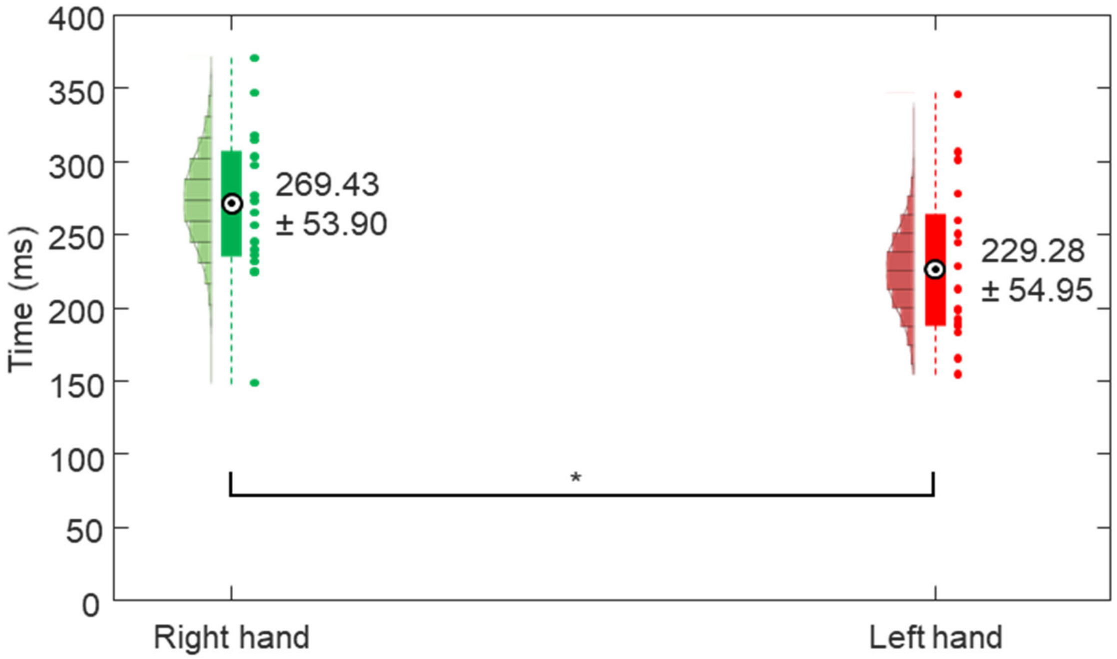

Figure S2. Statistical comparison of motor planning phase durations between hands. Boxplots present the group average (black circles) with values ± 1 standard deviation, individual means (colored dots), values percentile 25th to 75th range (colored rectangles), and limits for minimum to maximum values (dashed lines). The results for the right hand are in green, and the left hand in red. $Time_{PLAN}$ for the left hand was shorter than the right hand for both conditions (*$p<0.05$).

Part 3. Procedure for computing signal coupling (Corticomuscular and Intermuscular Coherence).

The procedure we applied to compute Corticomuscular Coherence (CMC) and Intermuscular Coherence (IMC) is based on Bigot et al (2011) method [30]. We have made minor variations in order to analyze events in a fixed-time window as we mentioned above. Here, we present a step-by-step explanation with an example between two signals.

1. Select two signals: EEG channel (C3 electrode) and EMG channel (right Anterior deltoid).

2. Select segments to analyze: 1-second window prior to the muscle contraction onset (Fig. S3 A).

3. Select repetitions to include: repetitions with duration within the range of the mean ± 1 standard deviation to mitigate the intra-subject variability.

4. Apply Continuous Wavelet transform of both segmented signals, for each repetition. In this step, WavCrossSpect toolbox by Bigot et al (2011) [30] was used with the parameters: 'Mother'=Morlet, 'nvoice'=5, 'J1'=100, 'wavenumber'=6, 'MaxScale'=default). This step provides an Auto-Spectrum (scalogram or time-frequency map) for every signal segment, for both EEG and EMG.

5. Compute 'Cross-Spectrum' between signals pair, for each repetition separately. This is a cross correlation between the two (EEG and EMG) auto-spectrums obtained on step 4.

6. Compute 'Mean Auto-Spectrum' for each signal separately (Fig S3 B), which is calculated as the point-by-point mean from the all power auto-spectrums of repetitions selected on step 3.

7. Compute 'Mean Cross-Spectrum' using 'Cross-Spectrum' from each signals pair (Fig S3 C), which is calculated as the point-by-point mean from every cross-spectrums computed on step 5.

8. Determine 'Significant Cross-Spectrum' (Fig S3 D, right panel), which is the points from 'Mean Cross-Spectrum' map that are above the threshold λ_α obtained with Equation S1, at level $\alpha = 0.05$, explained in detail by Bigot et al (2011) [30].

$$\lambda_\alpha = \frac{\rho_x \, \rho_y}{n} \left(-\log(\alpha/2) + \sqrt{-2n \log(\alpha/2)} \right), \qquad \text{(S1)}$$

where ρ_x and ρ_y are the largest eigenvalues of the empirical covariance matrices of both signals; and n is the number of repetitions.

9. Compute 'Magnitude-Squared Coherence' $R_{xy}^2(\omega, u)$, which is a normalized value from 0 to 1 (Fig S3 D, left panel), with Equation S2.

$$R_{xy}^{2}(\omega,u) = \frac{|S_{xy}(\omega,u)|^{2}}{S_{x}(\omega,u)\,S_{y}(\omega,u)}, \qquad \text{(S2)}$$

where $S_{xy}(\omega,u)$ is the 'Mean Cross-Spectrum'; $S_{x}(\omega,u)$ and $S_{y}(\omega,u)$ are the 'Mean Auto-Spectrums' of both signals.

10. Apply 'Significant Cross-Spectrum' matrix as a mask over 'Magnitude-Squared Coherence' to obtain normalized coherence only where the 'Mean cross-spectrum' was significant (Fig S3 E, left panel).

11. Boundary selection for the windows of interest within the time-frequency map depending of frequency and time interval. We decided to analyze the beta band (15 to 30 Hz) and two 500-millisecond windows (Fig S3 E, right panel).

12. Compute CMC as the single value (mean) of all values within each window of interest. Thus, we obtained one initial value ($CMC_{initial}$) and one final value (CMC_{final}). The latter is representative of the CMC into the PLAN phase.

13. The same procedure was performed between every EEG channel and the same EMG channel.

14. For IMC, we applied the same procedure using both anterior deltoid EMG signal.

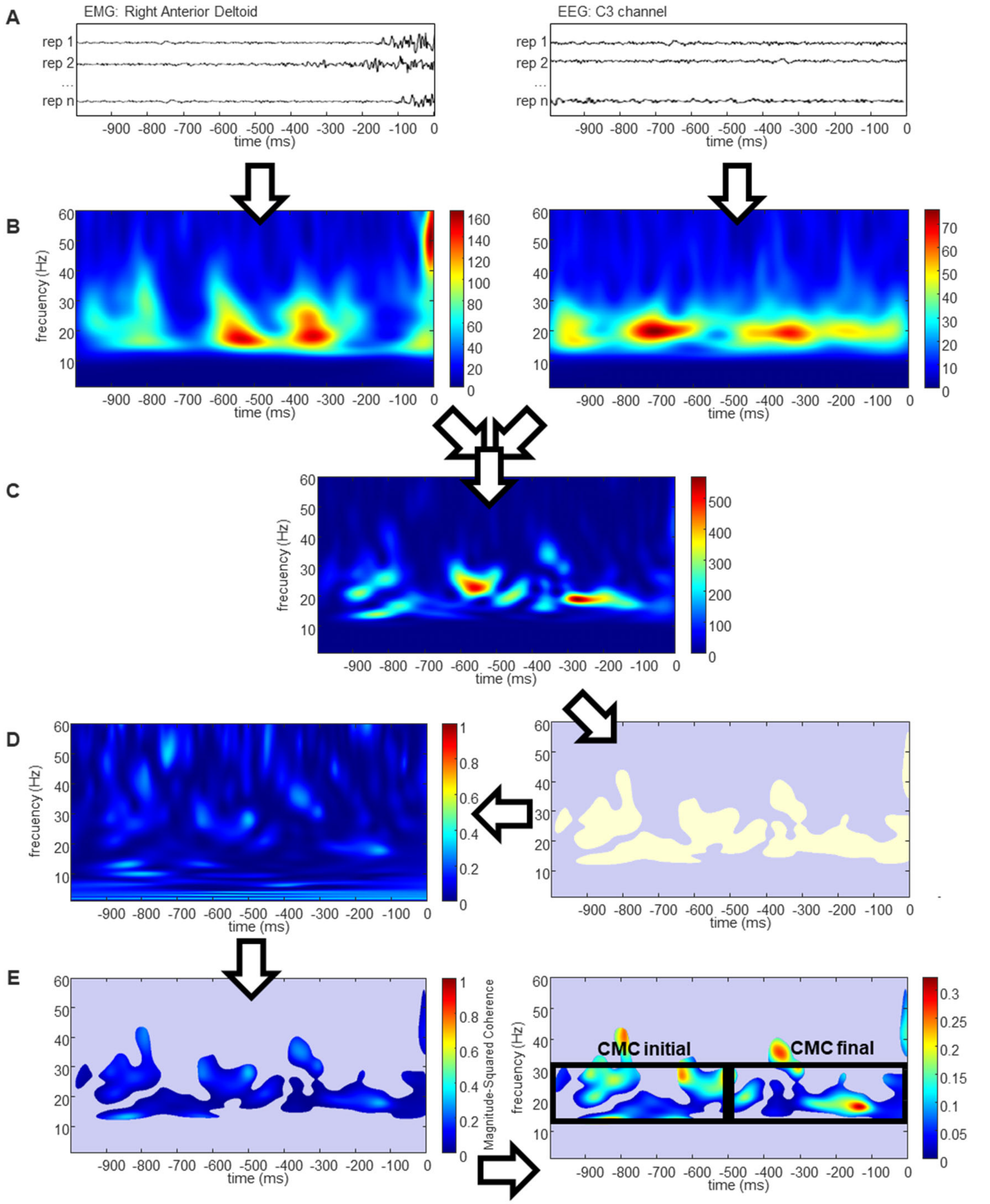

Figure S3. Summary for CMC compute procedure. (A) Selection of two signals (time series) with same length. (B) Compute 'Mean Auto-Spectrum' from the 'Wavelet Scalogram' of n repetitions for each signal separately. (C) Compute 'Mean Cross-Spectrum' from the 'Cross-Spectrums' from each signals pair (left column). (D, right panel) Determine 'Significant Cross-Spectrum'. (D, left panel) Compute 'Magnitude-Squared Coherence'. (E, left panel) ‘Magnitude-Squared Coherence’ masked by 'Significant Cross-Spectrum' (E, right panel) Coherence masked, color scale adjustment and delimitation for windows of interest to compute CMC value.
